# Editorial: Systems biology and data-driven machine learning-based models in personalized cardiovascular medicine

**DOI:** 10.3389/fcvm.2023.1320110

**Published:** 2023-10-27

**Authors:** Miguel Hueso, Noemí Rotllan, Joan Carles Escolà-Gil, Alfredo Vellido

**Affiliations:** ^1^ Department of Nephrology, Hospital Universitari Bellvitge, and Institut d’Investigació Biomèdica de Bellvitge-IDIBELL, L’Hospitalet de Llobregat, Spain; ^2^ BigData and Artificial Intelligence Group (BigSEN Working Group) from the Spanish Society of Nephrology (SENEFRO), Spain; ^3^ Institut d’Investigacions Biomèdiques (IIB) Sant Pau, Barcelona, Spain; ^4^ CIBER de Diabetes y Enfermedades Metabólicas Asociadas (CIBERDEM), Madrid, Spain; ^5^ Department of Computer Science, Universitat Politècnica de Catalunya (UPC), Barcelona, Spain; ^6^ Intelligent Data Science and Artificial Intelligence (IDEAI-UPC) Research Center, Barcelona, Spain

**Keywords:** personalized medicine, cardiovascular diseases, machine learning, artificial intelligence, systems biology

**Editorial on the Research Topic** Systems biology and data-driven machine learning-based models in personalized cardiovascular medicine

## 1. Introduction

The fields of health and medicine have joined the rest of the other branches of life sciences in adopting computerized systems, digital communication, information processing, and an overall data-centrism. Artificial intelligence (AI) has emerged as one of the current drivers for this data-centric approach, particularly in the form of machine learning (ML) and, more specifically, deep learning (DL), which is one of its most successful sub-families.

This brief editorial paper provides an introduction to the 16 papers that contributed to the current special issue on systems biology and data-driven ML-based models in personalized cardiovascular medicine. The collection comprises original research, systematic reviews, and meta-analyses, and we are confident that it will be of great interest to the readers of this journal. Cardiovascular medicine is in fact one of the most active medical areas for the application of ML and AI techniques (1). The breadth and variety of topics broached by the works in this collection bear testimony to such a reality. In the next paragraphs, we shall characterize these studies according to the cardiovascular problem addressed, the ML approaches adopted, and any other relevant characteristics of the data analysis workflows.

The studies conducted by Cornhill et al. and Dykstra et al. have reported on the predictions of heart failure hospitalization and atrial fibrillation based on similar cardiac magnetic resonance (CMR) information. In both cases, the CMR data are combined with electronic health record (EHR) features, in addition to supplementary information such as patient health questionnaires. In the study conducted by Peng et al. the authors address the problem of predicting all-cause in-hospital mortality for patients in the intensive care unit (ICU) with heart failure combined with hypertension. The data under analysis include gender, age, vital signs, laboratory tests, and comorbidities.

The study conducted by Ren et al. focuses on developing a predictive DL model for predicting the progression of cardiovascular disease (CVD), including coronary heart diseases, cerebrovascular diseases, congestive heart failure, and peripheral artery diseases in patients with diabetic kidney disease. The predictive model uses seven clinical variables, including age, smoking status, systolic blood pressure, total cholesterol, hemoglobin, high-density lipoprotein cholesterol levels, and urinary protein excretion. The best performing model, called DeepSurv, is used to develop an online tool for predicting CVD risk in patients with diabetic kidney disease.

Several papers (Cai et al., Guo et al., Li et al.) are devoted in predicting acute myocardial infarction (AMI). In the study conducted by Cai et al. the authors developed a predictive model for the risk stratification of acute kidney injury in patients with AMI using data from the Medical Information Mart for Intensive Care (MIMIC) IV database. Siva Kumar et al. in their study developed a quantitative electrocardiogram (ECG) risk score in conjunction with coronary artery calcification (CAC) to assess their ability in predicting major adverse cardiovascular events (MACE) in patients with at least one cardiovascular risk factor from the Community Benefit of No-charge Calcium Score Screening Program (CLARIFY) trial. A nomogram constructed by integrating the quantitative ECG risk score with CAC, age, and sex was found to be associated with MACE and demonstrated accurate discrimination between patients at high risk and those at low risk. The study conducted by Guo et al. identified the genes related with inflammation associated to the pathogenesis of AMI. This study performed, for the first time, a systematic analysis of biomarkers associated with the development from stable cardiovascular disease to AMI, specifically focusing on 5mC regulators. Interestingly, nine hub 5mC regulators were identified and validated by a robust model, leading to developing a diagnostic model that might be used to discriminate AMI from coronary artery disease. The study conducted by Li et al. focused on identifying the genes associated with heart failure induced by ischemic cardiomyopathy. Both papers utilized the data from the Gene expression Omnibus (GEO) and Genomic Spatial Event (GSE) databases. Weighted gene co-expression network analysis (WGCNA) is employed as a method in identifying potential functional modules. The CIBERSORT algorithm is used in characterizing immune cell infiltration.

In the study conducted by Kong et al. the authors employed a new proteomic assay platform called Olink multiplex cardiovascular disease III to assess the variations in protein expression in patients with acute phase atrial fibrillation who underwent cryoballoon ablation, radiofrequency balloon ablation, or radiofrequency ablation procedures. The pathway analysis revealed major changes in the cytokine–cytokine receptor interaction after the three different ablations, as well as in certain proteins associated with hemorrhage and coagulation. It should be noted that the scope of this exploratory study was rather constrained due to the small sample size and the focus on the proteins included in the Olink panel.

The problem of atrial fibrillation in patients with chronic obstructive pulmonary disease (COPD), using data from GEO and GSE databases, is addressed in the study conducted by Sun et al. These data were investigated using the WGCNA method and the STRING platform to construct a protein–protein interaction network. The CIBERSORT algorithm was used once again to characterize immune cell infiltration.

Shi et al. performed a retrospective observational study with 1,493 patients diagnosed with obstructive sleep apnea (OSA) admitted to the Department of Otorhinolaryngology—Head and Neck Surgery of the Second Affiliated Hospital of Xi’an Jiaotong University between October 2019 and December 2021. The authors used six different ML analyses and found that the gradient boosting machine (GBM) model was the best in assessing risk factors and predicting OSA-related hypertension. In addition to identifying several known risk factors, such as BMI, age/10, and minimum SaO_2_/10, the multivariate logistic regression and SHAP analysis also found that CT90/10OSA, a novel variable related to sleep disorder, exhibits a strong association with CVD, metabolic disorders, and cognitive impairment.

The study conducted by Song et al. provided a systematic review and meta-analysis that examined the use of 60 ML models in predicting cardiac surgery-associated acute kidney injury (CSA-AKI). The study included a total of 255,943 patients from 38 eligible studies and found that NNET and Extreme Gradient Boosting (XGBoost) are more effective in the early prediction of CSA-AKI compared with logistic regression (LR).

In another study, Zhou et al. used ML on the Genotype-Tissue Expression project (GTEx) database to find genes associated with sudden death (SD). The SD group included 88 blood samples from 69 donors with fast death of natural causes (0–1 h) and 17 donors with intermediate death (1–24 h). In this case, the authors employed two different ML algorithms, namely Least Absolute Shrinkage and Selection Operator (LASSO) and the Support Vector Machine with Recursive Feature Elimination (SVM-RFE), in order to reduce errors. Consequently, the analysis revealed a correlation between two specific genes, MYL2 and TNNT3, and the occurrence of SD.

One of the main barriers to the application of ML methods in clinical medicine is the difficulty of obtaining sizeable samples of harmonized, properly curated, and representative (multi-center, international) data. Many of the studies in this collection reflect this limitation and would require further validation in data-richer contexts in order to guarantee reproducibility. In any case, the reported circumstances are varied, as some works use original data, whereas others resort to existing publicly available databases. Among the former, for instance, the CMR data used in the studies conducted by Cornhill et al. and Dykstra et al. are derived from a single center and encompass a substantial number of cases. In the study conducted by Shi et al. a single-center retrospective design was used. However, more patients from multiple sources are required to validate the robustness and repeatability of their model. Among the latter, Peng et al. utilized data from several thousands of patients from the MIMIC-IV and the eICU Collaborative Research Databases. The meta-analysis conducted by Song et al. utilized an uncharacteristically large multi-center data sample of 255,943 patients. An interesting alternative approach was used in the study conducted by Feng et al. wherein the authors hypothesized that ML models can be effectively trained utilizing limited datasets by incorporating domain knowledge encoding.

The range of ML methods available to medical data scientists through open-access implementation is staggering. This issue reflects such variety: The most extreme example is the meta-analysis conducted by Song et al. where the performance of 60 ML models was compared. The studies conducted by Cornhill et al. and Dykstra et al. employed the Random Survival Forests as a statistical method. The research conducted by Peng et al. used a K-nearest neighbor method for missing data imputation. In addition, the study utilized the artificial neural networks (ANN), Naïve Bayes, and Random Forests (RF) as predictive models. In the study conducted by Cai et al. several analytical techniques such as RF, Bayesian analysis, SVM, XGBoost, Decision Trees, and LR to analyze the data. On the other hand, Li et al. utilized LASSO, RF, and SVM-REF for their analysis, while Guo et al. and Zhou et al. employed LASSO and SVM-RFE for their respective studies. The atrial fibrillation problem in Sun et al.’s study was analyzed using RF, SVM, XGBoost, and generalized linear models (GLM). Shi et al. employed LR, GBM, XGBoost, adaptive boosting (AdaBoost), bootstrapped aggregating (Bagging), and ANN for predicting OSA-related hypertension. XGBoost was also used in Li et al.’s study to assess the predictive value of a pressure recording analytical method for the duration of mechanical ventilation in children undergoing cardiac surgery, while LASSO and RF were also used in the study conducted by Lin et al. for the analysis of plasma protein profiling in patients with atrial fibrillation.

The lack of interpretability of ML models has recently emerged as a serious limitation in their implementation in medical settings (2). This topic has been investigated by Cai et al. and Shi et al. using the SHAP method to assess the relevance of input features. In addition, Lin et al. utilized an RF-related software package (in R) called *randomForestExplainer*.

This special issue offers an overview of the latest developments in AI and ML in the field of cardiovascular medicine. However, it is worth noting that future progress also points toward a more precise approach to treatment that takes into account individual differences in patient's genes, environmental factors, and lifestyle choices. This is a context in which AI and ML may help in improving diagnosis, drug discovery, and treatment personalization, perhaps with the help of new tools such as digital twins. The scalable storage of clinical data in data lakes that support fast multidimensional queries should enable data sharing and fuel clinical research. Finally, the emergence of large language models and generative AI, together with federated learning, may provide clinicians with powerful tools for bridging the gap between patients and devices, paving the way for interactive clinical decision support systems.
